# Co-administration of a DNA vaccine encoding the prostate specific membrane antigen and CpG oligodeoxynucleotides suppresses tumor growth

**DOI:** 10.1186/1479-5876-2-29

**Published:** 2004-09-09

**Authors:** Jiaqiang Ren, Li Zheng, Qi Chen, Hua Li, Lin Zhang, Hongguang Zhu

**Affiliations:** 1 Department of Pathology, Fudan University Shanghai Medical College, Yixueyuan Road 138, Shanghai, China

## Abstract

**Background:**

Prostate-specific membrane antigen (PSMA) is a well characterized prostate-specific tumor associated antigen. Its expression is elevated in prostate carcinoma, particularly in metastatic and recurrent lesions. These observations suggest that PSMA can be used as immune target to induce tumor cell-specific recognition by the host and, consequently tumor rejection. We utilized a DNA-based vaccine to specifically enhance PSMA expression. An immune modulator, such as CpG oligodeoxynucleotides which promote Th1-type immune responses was combined to increase the efficacy of tumor recognition and elimination.

**Methods:**

A eukaryotic expression plasmid pCDNA3.1-*PSMA *encoding full-length PSMA was constructed. C57BL/6 mice were immunized with endotoxin-free pCDNA3.1-*PSMA *alone or in combination with CpG oligodeoxynucleotides by intramuscular injection. After 4 immunizations, PSMA specific antibodies and cytotoxic T lymphocyte reactivity were measured. Immunized C57BL/6 mice were also challenged subcutaneously with B16 cells transfected with PSMA to evaluate suppression of tumor growth.

**Results:**

Vaccine-specific cytotoxic T lymphocytes reactive with B16 cells expressing PSMA could be induced with this treatment schedule. Immune protection was observed in vaccinated mice as indicated by increased tumor growth in the control group (100%) compared with the groups vaccinated with DNA alone (66.7%) or DNA plus CpG oligodeoxynucleotides (50%) respectively. Average tumor volume was smaller in vaccinated groups and tumor-free survival time was prolonged by the vaccination.

**Conclusion:**

The current findings suggest that specific anti-tumor immune response can be induced by DNA vaccines expressing PSMA. In addition, the suppression of in vivo growth of tumor cells expressing PSMA was augmented by CpG oligodeoxynucleotides. This strategy may provide a new venue for the treatment of carcinoma of prostate after failure of standard therapy.

## Background

Carcinoma of prostate is the most common cancer in males in America, ranking as the second most common leading cause of cancer-related deaths, just after carcinoma of the lung. In addition, the incidence and mortality of carcinoma of prostate are increasing in China. Although surgery and radiation therapy remain the primary choice for localized stage of carcinoma of prostate, there is no effective treatment for patients who develop recurrences or those who have metastatic disease at the time of diagnosis. Therefore, there is an urgent need for new types of treatment.

Strategies that stimulate the ability of the immune system to recognize and destroy cancer cells via selective killing mechanisms have shown promise in the treatment of cancer. DNA vaccines offer several potential advantages for the immunotherapy of cancer. Proteins encoded by DNA vaccines are expressed in the cytoplasm and presented through the endogenous processing pathway associated with MHC Class I molecules, thereafter leading to the activation of CD8 + cytotoxic T lymphocytes (CTL) [1,2], which act as effectors in the anti-tumor immune response. DNA vaccines are cost-effective since DNA is relatively simple to purify in a large quantities. Another intrinsic advantage consists in the presence in the plasmid itself of un-methylated CpG motifs (immunostimulatory sequences) that may act as a potent immunological adjuvant [3]. Thus, there is a good rationale for further development of DNA vaccines to immunize against antigens present on cancer cells.

Prostate specific membrane antigen (PSMA), a well-established prostate specific tumor associated antigen (TAA), is 100 kD type II transmembrane glucoprotein. It is predominantly expressed in the prostate gland, minimal levels of expression in brain tissue, jejunum and proximal kidney tubules [4,5]. Its expression is significantly elevated in carcinoma of prostate, particularly in metastastic disease and recurrent disease after hormone therapy fails [6,7]. These properties of PSMA propose it as an ideal target of anti-cancer vaccines.

A number of strategies are under evaluation to enhance the potency of DNA vaccines, some of which involves broad stimulation of the immune system using immunomodulatory agents. Synthetic CpG oligodeoxynucleotides have immunological effects similar to those seen with bacterial DNA and represent promising vaccine adjuvants, which promote T helper1 (Th1)-type immune responses [8]. Unmethylated CpG motifs are present at a much higher frequency in the genome of prokaryotes than eukaryotes. The release of unmethylated CpG DNA during an infection provides a 'danger signal' to the innate immune system, triggering a protective immune response that improves the ability of the host to eliminate the pathogen [9]. CpG oligodeoxynucleotides up-taken by B cells and plasmacytoid dentritic cells (pDCs), which express Toll-like receptror 9 (TLR9) [10,11] initiate an immune stimulatory cascade that culminates in the indirect maturation, differentiation and proliferation of T cells and natural killer (NK) cells[12,13]. Together, these cells secrete cytokines and chemokines that create a pro-inflammatory (IL-1, IL-6, IL-18 and tumor necrosis factor-α) and Th1-polarized (interferon-γ, and IL-12) immune *milieu *[14], which further facilitates the development of antigen-specific CTLs [15-17]. These effects indicate that CpG oligodeoxynucleotides could act as vaccine adjuvant. The present study was designed to test the therapeutic efficacy of a PSMA-based DNA vaccine in a mouse model of tumor cell implants expressing PSMA. In addition, the adjuvant role of CpG oligodeoxynucleotides to augment the potency of the constructed DNA vaccine was tested.

## Methods

### Mice and Cell lines

C57BL/6 mice (H-2b) were bred and kept under pathogen-free conditions. Male mice were used at 12 to 16 weeks of age. All animal experiments were performed in an approved protocol and in accordance with recommendations for the proper care and use of laboratory animals.

The murine melanoma B16 cell was purchased from the Type Culture Collection of the Chinese Academy of Sciences and cultured in RPMI-1640 medium (Life Technologies, Gaithersburg, MD) supplemented with 10% (v/v) heat-inactivated fetal bovine serum (Hyclone, Logan, UT), penicillin G (100 U/ml), and streptomycin (100 μg/ml).

The COS-7 cell line was cultured in DMEM medium (Life Technologies) supplemented with 10% (v/v) heat-inactivated fetal bovine serum, sodium pyruvate (1 mM), penicillin (100 U/ml) and streptomycin (100 μg/ml).

### Antibodies and reagents

The monoclonal antibody 4A12 specific for an extra-cellular epitope of PSMA was previously described [18]. The β-actin-specific monoclonal antibody was purchased from Sigma Chemical Co (St. Louis, MO). FITC-labeled goat anti-mouse IgG was purchased from Santa Cruz Biotech (Santa Cruz, CA). Recombinant murine IL-2 was purchased from PeproTech (Rocky Hill, NJ). ConA and mitomycin C were purchased from Sigma Chemical Co.

CpG oligodeoxynucleotides 1826 chosen according to published data [19-21] had the following sequence TTCATGACGTTCCTGACGTT (CpG motifs were shown underlined) with the backbone phosphorothioate stabilized. CpG oligodeoxynucleotides were synthesized by Sangon (Shanghai, China), reconstituted in sterile pyrogen-free water and diluted in phosphate buffered saline for in vivo injections.

### Transient transfection of COS7 cells

The eukaryotic expression plasmid pCDNA3.1-*PSMA *encoding full-length PSMA was constructed by cloning the BamH I /Xho I fragment of pBluescipt-*PSMA *as described previously [18] into the pCDNA3.1 vector (Invitrogen Corp, Carlsbad, CA) cut with identical endonuclease.

COS-7 cells were transfected with pCDNA3.1-*PSMA *or an empty (mock) vector by the mediation of liposome Tfx-20™ (Promega, Madison, WI) according to the manufacturer's instructions. Briefly, COS-7 cells were cultured in six-well tissue culture plates with a coverslip in each well and grown to 50–70% confluence. 1.5 μg plasmid DNA was mixed with 4.5 μl Tfx-20™ and diluted in 1000 μl serum-free DMEM medium before addition to cells. After 20 minutes of incubation, DNA-liposome complex was added to the cells and incubated for 6 hours at 5% CO2, 37°C. Complete DMEM medium containing 10% fetal bovine serum was added to the cells and incubated overnight, and then the medium was replaced with complete DMEM medium. After 2 days, the cells were fixed in cold acetone for 10 minutes at 4°C followed by extensive washing with phosphate buffered saline. The cells were incubated with anti-PSMA monoclonal antibody 4A12 for 1 hour at 37°C, subsequently incubated with FITC-labeled goat anti-mouse IgG (1:40) for 1 hour at 37°C. After thorough washing, the coverslips were mounted and observed with a fluorescence microscope.

### Stable transfection of B16 melanoma cells with *PSMA *plasmid

The B16 murine melanoma cells were transfected with 2 μg of pCDNA3.1-*PSMA *or empty vector by the mediation of 6 μl liposome Tfx-20™ as above. After 2 days of culture, the cells were reseeded into a 10 cm-dish and cultured for other 2 days, complete RPMI-1640 medium containing 1000 μg/ml G418 (Life Technologies) was added to the culture. After 20 days of selection, all non-transfected cells died and discrete clones were visible in transfected cells. These clones were expanded in the presence of 400 μg/ml G418, positive cells expressing PSMA were identified as follows.

### Detection of *PSMA *mRNA by Reverse Transcriptase-PCR

Total RNA was extracted from mock-transfected or transfected B16 cells using Trizol (Life Technologies) and dissolved in RNase free water. 2 μg of total RNA was transcribed into cDNA using AMV reverse transcriptase (Promega). Briefly, the total RNA was mixed with 1 μl oligo (dT) primers (0.1 μg/ μl), 4 μl RT Buffer (5×), 2 μl dNTPs (10 mM), 1 μl AMV reverse transcriptase and diethypyrocarbonate-treated water to a final volume of 20 μl. The cDNA synthesis was performed using the following PCR parameters: 37°C for 1 hour then 10 minutes at 95°C. Synthesized cDNA was used as template for PCR. The sequence of the primers used were 5'- CGAGGAGGG ATGGTG TT-3' (forward) and 5'-TGTTGTGGCTGCTTGAG-3' (reverse). PCR was carried out in a 10 μl aliquot containing 0.5 μl cDNA, 0.5 μl each primer (10 μM), 1 μl dNTPs (2 mM), 1 μl Taq buffer (10×), 0.8 μl MgCl_2 _(25 mM), 1 unit of Taq. The PCR reaction conditions included 5 minutes of initial denaturation at 94°C followed by 30 cycles of 30 seconds at 94°C, 1 minute at 62°C, 1 minute at 72°C and 10 minutes of final extension at 72°C. The 358 bp fragment was resolved on 2% agarose gel. GAPDH was also detected as internal reference.

### Detection of PSMA protein by Western Blot

The transfected and mock-transfected B16 cells were harvested and lysed with lysis buffer (50 mM NaCl, 0.01 M Tris-Cl (pH8.0), 5 mM EDTA, 0.5% NP-40, 1 mM phenylmethylsulfonyl fluoride, 1 μg/mL aprotinin, 1 μg/mL leupeptin) for 30 min at 4°C, cell debris were removed by centrifugation. Cell lysates were heated at 100°C for 3 minutes, the samples were loaded on 6% SDS-PAGE for electrophoresis. After electrophoresis, the proteins were transferred to polyvinylidene difluoride membranes (Amersham Pharmacia Biotech, Piscataway, NJ) using semi-humid transferring system (Bio-Rad, Hercules, CA). The polyvinylidene difluoride membranes were blocked with Tris-buffered solution containing 5% (w/v) non-fat milk for 1 hour at room temperature. For detection of protein, the polyvinylidene difluoride membranes were probed with anti-PSMA monoclonal antibody 4A12 and monoclonal antibody to β-actin (1:50) respectively for 1 hour at room temperature then overnight at 4°C, after then the membranes were incubated with horse anti-mouse IgG-HRP conjugate (1:500, Vector, Burlingame, CA) for 1 hour at 37°C, ABC complex (1:500, Vector) for 1 hour at 37°C subsequently. The bands were visualized with 3,3'-diaminobenzidine substrate solution (5 mg diaminobenzidine dissolved in 10 ml Tris-buffered solution, 10 μl 30% hydrogen peroxide).

### DNA vaccination of C57BL/6 mice

Plasmids pCDNA3.1-*PSMA *and pCDNA3.1 were purified with EndoFree plasmid Maxi Kit (Qiagen, Valencia, CA). Three groups including 6 mice each were immunized: DNA vaccination group, CpG oligodeoxynucleotides and DNA vaccine co-administration group (hereafter referred to as CpG+DNA vaccination) and control group receiving empty plasmid. All mice were injected with 0.25% lidocaine in the quadriceps femoris muscle 3 days before vaccination in order to improve the uptaking of plasmids by muscles. The mice then received bilateral intramuscular injection with 50 μg of plasmid in the regenerating muscles. Mice in the DNA vaccination group were immunized with endotoxin-free pCDNA3.1*-PSMA*, mice in CpG+DNA vaccination group were further immunized with 25 μg of CpG oligodeoxynucleotides in the same location 3 days after DNA plasmid immunization, as control, mice were injected with pCDNA3.1 plasmid. All mice were boosted every 4 weeks for 3 times.

### Measurement of PSMA specific serum antibodies

Two weeks after the last immunization, the mice were bled and serum antibodies were measured by solid phase enzyme-linked immunosorbent assay (ELISA). Briefly, bacterially expressed fusion protein containing PSMA-derived fragment was coated on 96-well plates. The plates were blocked with 5% bovine serum albumin in phosphate buffered saline overnight at 4°C. The sera from C57BL/6 mice were serially diluted in phosphate buffered saline with 5% bovine serum albumin and then 100 μl of diluted serum was added into each well. The plates were incubated at 37°C for 1 hour, 100 μl of a 1:3000 dilution of goat anti-mouse IgG-HRP conjugate (Jackson ImmunoResearch, West Grove, PE) was added into each well and incubated for 1 hour at 37°C, then 100 μl tetramethyl benzidine (TMB) chromagen/substrate solution (0.1 mg/ml TMB, 0.1 M citric acid buffer pH6.0, 4 μl 30% hydrogen peroxide per 10 ml) was added to each well. The plates were read and the absorbance at 450 nm (A450) was measured by microplate reader.

### Cytotoxic T Lymphocyte (CTL) Assay

Two weeks after the last immunization, mice were sacrificed. Their spleens were removed and teased apart in serum-free RPMI-1640 media, the lymphocytes were collected and cultured in RPMI-1640 medium supplemented with 10% heat-inactivated fetal bovine serum, 50 IU/ml recombinant murine IL-2 and 2 μg/ml ConA for 2 days. B16 stimulator cells expressing PSMA (B16-PSMA) were prepared in complete RPMI-1640 medium containing 50 μg/ml mitomycin C for 1 hour at 37°C. Two × 10^7 ^lymphocytes (responders) were incubated in 6-well plates with 2 × 10^6 ^stimulator cells in the presence of 50 IU/ml recombinant murine IL-2 and 2 μg/ml ConA. After 6 days of culture, the re-stimulated cells were harvested and separated from the dead cells. Target cells (B16-PSMA) and re-stimulated lymphocytes (effector cells) were resuspended in phenol red-free RPMI-1640 medium supplemented with 5% new born calf serum, 5 × 10^3 ^target cells and various number of effector cells were added into individual flat-bottom wells in 96-well plates. The cells were incubated at 37°C overnight. 50 μl per well of supernatant was transferred to fresh 96-well plates. CTL reactivity was assessed by measuring lactate dehydrogenase (LDH) release using a Cytotox 96 assay kit (Promega). Controls were setup on each plate for spontaneous LDH release by target and effector cells. A parallel experiment using B16 cells transfected with pCDNA3.1 as target cells was also performed to test the specificity of lysis. All experiments were performed in triplicate. Percent lysis was calculated according to manufacturer's instructions.

### Subcutaneous transplantation of tumor cells

C57BL/6 mice were divided into 3 groups and immunized as above. B16-PSMA cells were trypsinized and resuspended in phosphate buffered saline, 2 × 10^5 ^cells were subcutaneously injected into the left lateral flank of mice. The time to the development of tumor was recorded. After tumors became detectable, their volume was measured two-dimensionally with a caliper along the longest axis (x) and the axis perpendicular to the longest axis (y) every second days. The volume of tumors was estimated by the following formula:

Volume = π/6 × x × y^2^

After 26 days, when tumor reached 20 mm in their largest axis, the mice bearing tumors were sacrificed. Tumors were removed and weighed.

### Data Analysis

The data from ELISA and CTL assays are expressed as means ± SD and are representative of at least three different experiments. Comparisons between individual data points were made using ANOVA or student's t-test. In the tumor challenge experiment, the primary endpoint was time of tumor appearance. Tumor-free survival time was compared by the Kaplan-Meier method and log-rank statistic. *P *< 0.05 were considered significant.

## Results

### Expression of plasmid *pCDNA3.1-PSMA *in COS7 cells

**Figure 1:**
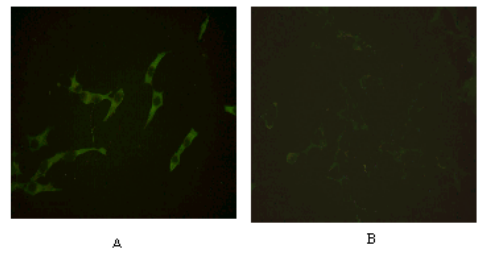
**Immunofluorescence staining of COS7 cells. **The COS7 cells were transfected with either pCDNA3.1-*PSMA *or empty pCDNA3.1 and fixed with cold aceton. Fixed cells were incubated with a anti-PSMA monoclonal antibody 4A12, stained with a goat anti-mouse immunoglobulin-FITC conjugate, PSMA immunoreactive cells were visualized with a fluorescent microscope (×40). Cytoplasmatic reactivity was found in COS7 cells transfected with pCDNA3.1-*PSMA *(A) but not in COS7 cells transfected with pCDNA3.1 (B).

To confirm the expression of PSMA in mammalian cells, plasmid pCDNA3.1*-PSMA *was introduced into COS7 cells. The cells were the incubated with anti-PSMA monoclonal antibody and goat anti-mouse IgG-FITC conjugate. The immunofluorescence assay demonstrated that the reactivity was present in the cytoplasm of COS7 cells transfected with pCDNA3.1-*PSMA *but not in mock-transfected cells, thus indicating that pCDNA3.1*-PSMA *could express protein in mammalian cells (Figure 1). We considered that plasmid pCDNA3.1 containing the Simian virus 40 (SV40) origin of replication were rapidly amplified in COS7 cells, which constitutively express SV40 large T antigen (T-Ag), so pCDNA3.1-*PSMA *underwent multiple rounds of duplication within one cell generation. A large amount of protein therefore was expressed and could not be delivered completely through the transporting machinery to cytomembrane, this may explain why the reactivity was found in cytoplasm.

### Detection of *PSMA *mRNA

**Figure 2:**
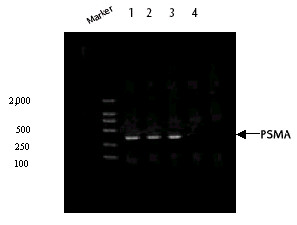
**Detection of *PSMA *mRNA by RT-PCR. **The murine melanoma B16 cells were transfected with either pCDNA3.1-*PSMA *or empty pCDNA3.1 and selected by G418. After 20 days of selection, clones resistant to G418 were acquired. Total RNA was extracted, and *PSMA *mRNA was detected by RT-PCR. 3 clones of B16 cells transfected with pCDNA3.1-*PSMA *were positive for *PSMA *mRNA, while B16 cells transfected with pCDNA3.1 were negative. Lanes 1, 2 and 3, B16 clones transfected with pCDNA3.1-*PSMA*; lane 4, B16 clones transfected with pCDNA3.1.

The transfected and mock-transfected B16 murine melanoma cells were selected by G418 and, after 3 weeks of selection, clones resistant to G418 were obtained. Total RNA was extracted and reverse-transcribed into cDNA to be used as template for PCR detection of *PSMA *mRNA. A 358 bp band was present in 3 B16 clones transfected with pCDNA3.1-*PSMA *but not in clones transfected with pcDNA3.1 (Figure 2). GAPDH were detected in all samples (data not shown).

### Detection of PSMA protein

**Figure 3:**
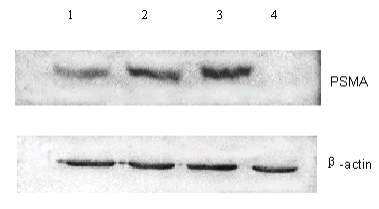
**Detection of PSMA protein by Western Blot. **Total cell lysates were harvested and presence of PSMA protein was detected by anti-PSMA monoclonal antibody 4A12. A 100 kD band was identified in 3 clones with detectable *PSMA *mRNA but not in B16 cells transfected with empty vector (A). Lane1, 2 and 3, B16 cells transfected with pCDNA3.1-*PSMA*. Lane 4 B16 cells transfected with pCDNA3.1. β-actin was used as reference (B).

The lysates from 3 clones with detectable *PSMA *mRNA were resolved by 6% SDS-PAGE followed by immunoblotting. A predicted 100 kD band was identified in the 3 clones but not in mock-transfected B16 cells (Figure 3). One of the clones was designated as B16-PSMA and used as target cells for cytotoxic T lymphocytes (CTL) assay or in tumor challenge experiment. A B16 cell line transfected with pCDNA3.1 (referred to as B16-pCDNA) was also obtained and used as negative control in CTL analysis.

### Measurement of PSMA specific serum antibodies

**Figure 4:**
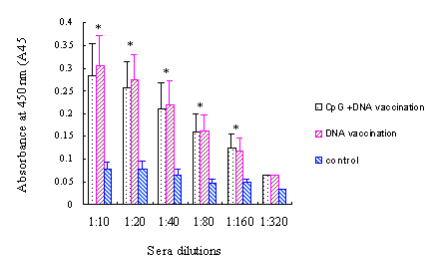
**Measurement of PSMA specific serum antibodies. **Mice were immunized 4 times at 4 weeks intervals by intramuscular injection with pCDNA3.1-*PSMA *or pCDNA3.1, antibody titers were measured by ELISA. Sera were serially diluted and measured individually. The experiment was performed in triplicate. Shown is the average antibody titer (n = 6) with standard errors. *P *valure was calculated by ANOVA. Antibody titer was similar in DNA vaccination group and CpG + DNA vaccination group, no antibody was detected in control group. * indicated the difference between control group with other two groups was statistically significant (*P *< 0.05).

ELISA was used to measure PSMA specific serum antibodies in C57BL/6 mice. All mice immunized with pCDNA3.1-*PSMA *generated low titers of antibodies, while PSMA specific antibodies were not detected in control group. When sera were diluted at 1:10, 1:20,1:40,1:80,1:160, the differences between control group and other two groups were all statistically significant (*P *< 0.001). However, titers were similar between the DNA vaccine and the CpG+DNA vaccination groups (*P *> 0.05), suggesting that CpG oligodeoxynucleotides did not augment the antigen-specific humoral immunity (Figure 4). The experiment was repeated 3 times with sera from independently immunized mice yielding comparable results.

### Cytotoxic T Lymphocyte (CTL) Assay

**Figure 5:**
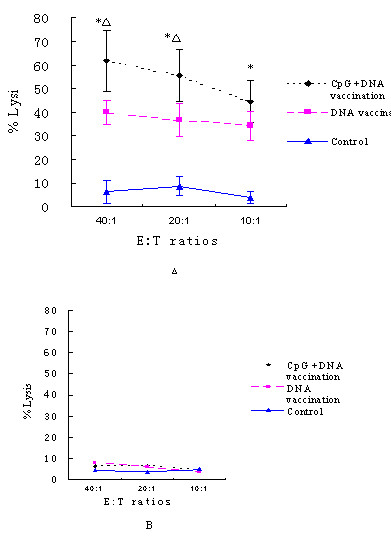
**Cytotoxic T Lymphocyte (CTL) Assay. **Splenocytes from C57BL/6 mice were re-stimulated for 6 days with mitomycin C-treated B16-PSMA cells. Cytotoxicity was measured by LDH release assay. The experiment was carried out in triplicate. Shown is the average CTL (n = 6) with standard errors. When B16-PSMA cells were used as target cells, specific lysis was found in DNA vaccination group but not in control group. The CTL reaction was enhanced by CpG oligodeoxynucleotides(Fig. 5A). However, when the mock-transfected B16-pCDNA cells were used as target cells, specific lysis was not observed in all groups (Fig. 5B). * indicated the difference between control group with other two groups was statistically significant (*P *< 0.05); △ indicated the difference between DNA vaccination group and CpG + DNA vaccination group was statistically significant (*P *< 0.05).

Splenocytes from C57BL/6 mice were re-stimulated for 6 days with mitomycin C-treated B16-PSMA cells. Cytotoxicity was measured by LDH release from attacked B16-PSMA cells or B16-pCDNA cells. Splenocytes from mice in the DNA vaccination group exhibited specific lysis against B16-PSMA, whereas those from mice in the control group did not acquire killing activity. The differences between control group and the other two groups were statistically significant at E:T ratios of 40:1, 20:1, 10:1 (*P *< 0.01). More importantly, CTL reactivity was significantly enhanced in mice treated with CpG oligodeoxynucleotides compared with DNA vaccine alone at E:T ratios of 40:1, 20:1 (t = 9.737, *P *< 0.001; t = 2.14,*P *= 0.021 respectively) (Figure 5A). However, specific lysis was not observed in all groups when B16-pCDNA cells were used as target cells (Figure 5B).

### Suppression of Tumor Growth in Tumor-bearing Mice by pCDNA3.1-*PSMA*

**Figure 6:**
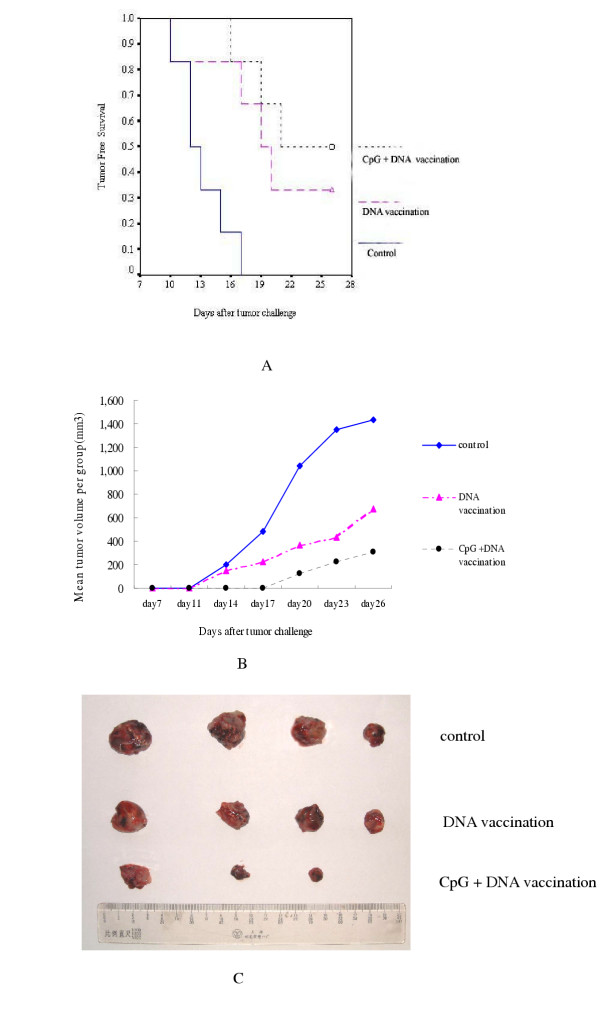
**Suppressive Effects of DNA vaccine to Tumor Growth. **C57BL/6 mice (n= 6/group) were vaccinated with pCDNA3.1-*PSMA *or empty vector, and then challenged with B16-PSMA cells. Kaplan-Meier curves showed the tumor-free survival time of mice was 19.67 ± 2.24 days in DNA vaccine group, 22.33 ± 1.61 days in CpG + DNA and 13.17 ± 1.01 days in control group respectively (Fig. 6A). Some mice still developed tumors after pCDNA3.1-*PSMA *and CpG oligodeoxynucleotides vaccination, but the individual tumors grew much more slowly than those in control group (Fig. 6B). Two mice in control group died before the termination of the experiment, all mice bearing tumor were sacrificed and tumor tissues were removed, the volumes of tumors in control group were larger than those in other two groups (Fig. 6C).

C57BL/6 mice (n= 6/group) were vaccinated with pCDNA3.1-*PSMA *or empty vector, and then challenged with B16-PSMA cells. Protection was observed in pCDNA3.1-*PSMA *vaccinated mice with decrease of tumor incidence. After 26 days, all mice in the control group developed tumors while 2 (2/6) and 3 tumor-free mice (3/6) were observed in the DNA and in the CpG + DNA vaccination groups respectively. Kaplan-Meier curves showed that the tumor-free survival interval was 19.67 ± 2.24 days in the DNA vaccination group, 22.33 ± 1.61 days in the CpG +DNA vaccination group and 13.17 ± 1.01 in the control group (Fig. 6A). The difference between DNA vaccination group and control group was statistically significant (*P *= 0.0161), so was the difference between CpG+DNA vaccination group and control group (*P *= 0.0016). Tumor-free survival time was longer in CpG+DNA vaccination group than DNA vaccination group, but the difference was of no statistical significance (*P *= 0.49). This observation may be associated with the small number in each group.

Although mice developed tumors after pCDNA3.1-*PSMA *vaccination, the individual tumors were consistently smaller than those in the control group and the analysis of tumor growth kinetics indicated that the tumor growth was significantly slower in the CpG +DNA vaccination group compared to the other two groups (Fig. 6B).

The volume of the tumors in the control group was consistently larger than in other two groups (Fig. 6C) and the average tumor weight was 2.28 ± 0.51 g in the DNA vaccine group, 1.10 ± 0.70 g in CpG + DNA group and 4.75 ± 0.66 g in the control group. The differences between control group and DNA vaccination group, CpG +DNA vaccination group were statistically significant (t = 5.92, *P *= 0.001; t = 7.062, *P *= 0.001 respectively). Moreover, the difference between DNA vaccination group and CpG +DNA vaccination group was statistically significant (t = 2.588, *P *= 0.049).

## Discussion

Although treatments are available for organ-confined carcinoma of prostate, there is no effective approach to treat recurrent disease after androgen deprivation therapy fails. New approaches are required to treat this incurable disease. DNA vaccination enables maintenance of tumor antigen expression at the vaccination site and results in immune responses in the host, therefore, shedding light on the treatment of cancer. It has been reported that tumor growth is suppressed when tumor cells are implanted in mice previously immunized with DNA vaccines encoding tumor antigens [22-25].

PSMA is a well-defined prostate-restricted tumor associated antigen whose expression is significantly elevated in carcinoma of prostate, especially in advanced stages. The expression of PSMA is down-regulated by androgen, after androgen deprivation therapy, its expression is strongly elevated[6,26-28], Thus, PSMA is a potential target for the immunotherapy to carcinoma of prostate. Several PSMA-based vaccines had been developed and it has been observed in a phase II trial utilizing MHC Class I-restricted peptides that PSMA can induce immune responses in patients with advanced carcinoma of prostate and alleviate the disease [29-31]. This observation suggests PSMA as an appropriate target of active-specific immunization against carcinoma of prostate. However, standard methods of protein/epitope preparations often coupled to the adoptive transfer of antigen presenting cells are labor-intensive decreasing the widespread use of vaccines in the general cancer patient population.

The purpose of our work was to delineate new ways to induce immune responses by DNA vaccination. In this study, all mice immunized with DNA vaccine expressing PSMA generated PSMA specific antibodies at a low level, which may result from the small amount of antigen expressed by plasmid in vivo. What is noteworthy is that all immunized mice developed CTL reactivity to B16-PSMA which led to suppression of tumor growth. In addition, although some tumors developed in some treated mice, they were consistently smaller in the control group. These findings suggest that DNA vaccines expressing PSMA could elicit immune response against tumor cells expressing the target molecule.

Although DNA vaccines provide a convenient and effective approach to elicit cellular immunity, clinical outcomes have not been satisfactory, mainly because tumor-specific CTL elicited by the vaccines are insufficient to suppress cancer progression. CD8+ CTLs constitute one of the most important arms of the immune system, exhibiting the capacity of recognizing and destroying cancerous cells[32,33]. A variety of approaches are under evaluation to activate CD8+ CTLs, to that end, vaccines need to be administered in combination with adjuvants of which the most commonly used in experimental models is incomplete Freud's adjuvant (IFA). However, this adjuvant is not widely used in human vaccination protocols due to its undesirable side effects, such as erythema and induration at the injection site, in addition, IFA functions mainly to promote humoral immunity. For these reasons, alternative potent and safe adjuvants need to be identified to enhance cellular immune response against cancer [34,35].

Synthetic CpG oligodeoxynucleotides represent a promising adjuvant. The predominant effect of CpG oligodeoxynucleotides exposure is the promotion of Th1-type immune responses. Professional antigen presenting uptake CpG oligodeoxynucleotides and become activated with increased expression of MHC and co-stimulatory molecules [36-38] that promote antigen presentation to naïve T cells. In addition, dentritic cells are stimulated to secret Th1-biased cytokines, such as interferon-γ and IL-12 particularly desirable in cancer immunotherapy [39,40]. Therefore, CpG oligodeoxynucleotides may be useful vaccine adjuvants.

CpG oligodeoxynucleotides 1826 is a potent enhancer of Th1-type immune responses and may benefit anti-cancer therapy [41-43]. We, therefore, hypothesized that the administration of these CpG oligodeoxynucleotides should enhance the cellular immunity elicited by DNA vaccines. However, co-adminstration of CpG oligodeoxynucleotides and DNA vaccines inhibit each other activity because CpG oligodeoxynucleotides may compete with plasmid uptake by antigen presenting cells. Furthermore, IFN-γinduced by CpG oligodeoxynucleotides could inhibit the activity of the CMV promoter utilized by eukaryotic expression vector, thus decreasing antigen expression. To examine the efficacy of CpG oligodeoxynucleotides as vaccine adjuvants, we injected them at the DNA injection site 3 days after vaccination rather than simultaneously. This strategy was based on a previous observation that transfected cells reach maximum yield of antigen expression between day 2 and 3 after vaccination. In this context, delivering the CpG oligodeoxynucleotides at the time of maximal antigen expression may be crucial to optimize the immunogenic boost [44,45].

Consistent with previous reports, this study suggests that CpG oligodeoxynucleotides enhance cellular immunity. The activity of CTL against PSMA expressing cells in the CpG +DNA vaccination group was significantly higher than in the DNA vaccination group. Furthermore, tumor challenge experiments demonstrated a potentiation of the suppressive effects on the growth of tumor cells expressing PSMA. These findings indicate that CpG oligodeoxynucleotides should be a powerful adjuvant in the context of DNA-based vaccination.

## Conclusions

In this study, we designed a DNA vaccine expressing prostate specific membrane antigen (PSMA) and utilized CpG oligodeoxynucleotides to promote Th1-type immune response. We discovered that the constructed vaccine generated anti-tumor reactivity against malignant cells expressing PSMA that was enhanced by CpG oligodeoxynucleotides co-administration. This strategy may provide a new venue for the treatment of carcinoma of prostate, particularly for recurrent disease after hormone therapy fails.

## Competing interests

None declared.

## Authors' contributions

R.J.Q participated in the design of the study and carried out plasmid DNA transfection, RT-PCR, immunofluorescence assay, DNA vaccination, lymphocyte stimulations, cytotoxicity assays, and completed the preparation of the manuscript. Z.L participated in the design of the study and carried out the construction of the expression plasmid, western blotting, and histological analysis and assisted in the preparation of the manuscript. C.Q carried out cell culture, L.H carried out RNA extraction, Z.L carried out ELISA. Z.H.G conceived of the study, participated in its design and coordination, and helped draft the manuscript. All authors read and approved the final manuscript.
